# Forced Dilatation in the Treatment of Hemorrhoids

**Published:** 1886-11

**Authors:** 


Forced Dilatation in the Treatment of Hemorrhoids.—The indications for the employment of forced dilatation are pain and hemorrhage, the operation being an excellent means of combating these accidents of hemorrhoids. It should not be employed in cases of prolapsus without contraction. Dilatation sometimes gives permanent relief, although the disease generally returns, when the operation may be repeated.

